# A Case of Phlebectasia in a Child Presenting With Neck Mass

**DOI:** 10.7759/cureus.48987

**Published:** 2023-11-18

**Authors:** Neha D Shetty, Rajasbala Dhande, Pratap Parihar, Bhavik S Unadkat, Nikita Bora, Prasad Desale

**Affiliations:** 1 Radiodiagnosis, Datta Meghe Institute of Higher Education and Research, Wardha, IND

**Keywords:** pediatric neck masses, neck anterior neck mass, usg jugular vein, congenital venous ectasia, internal jugular phlebectasia, internal jugular vein abnormality

## Abstract

Jugular phlebectasia is an enlargement of the jugular vein that manifests as a soft, cystic lump in the neck which can be compressed, becomes prominent on crying or straining and disappears on rest. It needs to be distinguished from laryngocele, neck cysts, and tumours that can also develop with straining. We report a case of a seven-year-old boy presenting with a cervical cystic mass. Comparable computed tomography and ultrasonography findings helped identify the pathology.

## Introduction

Arterial aneurysms, which result from multifactorial vascular deterioration and often manifest between the sixth and eighth decades of life, are frequently seen in adult clinical practice [1]. Venous aneurysms are relatively uncommon, and therefore, congenitally weak vessel walls typically cause them rather than degenerative processes. They more frequently affect children than adults [2,3]. Due to the relatively wide range of differential diagnoses in the pediatric age group, neck masses in children might present a diagnostic challenge. The differentials are narrowed down when a neck mass enlarges during a Valsalva manoeuvre. A tumour or cyst arising from the superior mediastinum, branchial cleft cyst, jugular vein phlebectasia (JVP), or laryngocele is a possible differential to be considered [4].

Phlebectasia is the term for an abnormal fusiform vein enlargement. Compared to lower limb venous aneurysms, which can in adults lead to pulmonary thromboembolism, upper extremities and cervical vein venous aneurysms are more prevalent and tend to be asymptomatic. Ultrasound scanning with duplex mode is typically sufficient to diagnose venous aneurysms, but other imaging techniques can be used to improve the morphologic assessment, including 3D ultrasound and cross-sectional imaging such as computed tomography (CT), magnetic resonance imaging with magnetic resonance venography, and catheter-directed venography [5].

## Case presentation

A seven-year-old boy visited the tertiary care centre with a right-sided neck mass, which he had since the boy was three years old and not associated with pain. The swelling was non-progressive in nature and apparent only while the boy strained or cried. The swelling disappeared completely at rest. The boy had no complaints of cough, hoarseness in the voice, or fever. He did not complain of breathing or swallowing problems. The boy’s guardians mentioned no trauma to the neck in the past or any infections affecting the cervical region.

**Figure 1:**
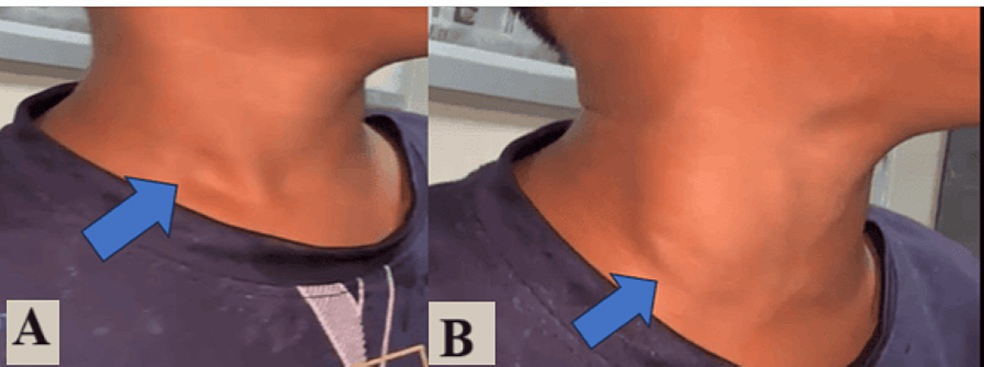
Lateral view of the right side of the neck (A) without straining and (B) with straining (Valsalva technique) showing no visible swelling at rest and visible swelling after straining (blue arrow)

A general physical examination revealed no evidence of pallor, icterus, or cyanosis and no signs of oedema or lymphadenopathy were observed. He appeared healthy in general physical examination. At rest, the patient’s neck did not reveal any evident mass. On straining and while performing the Valsalva manoeuvre, a 4 × 3 cm ovoid swelling appeared on the right side of the neck in the anterior triangle of the neck just in front of the lower two-thirds of sternocleidomastoid muscle on the right side (Figure 1).

**Figure 2:**
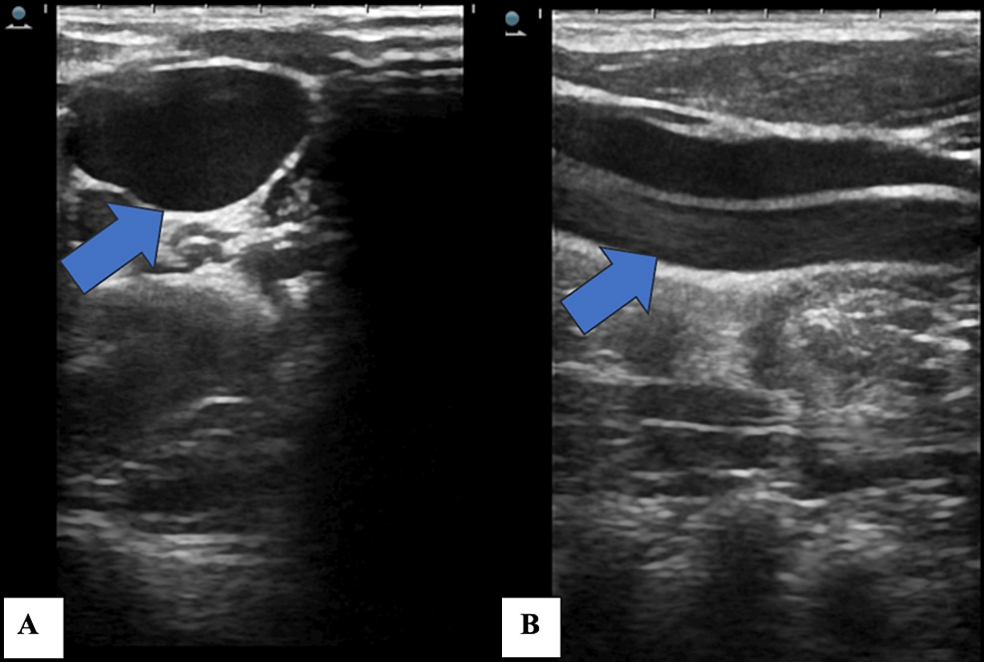
Grey-scale ultrasonography demonstrating dilated internal jugular vein (blue arrow) on the right side in (A) axial and (B) transverse views

While palpating the swelling, it was non-tender, soft, cystic and non-mobile. The overlying skin had no change in colour or rise in temperature. No visible pulsations were noted over the swelling. The rest of the examination of the tympanic cavity, nasal cavity, and pharynx was unremarkable. The boy was advised to undergo ultrasonography of the neck. It revealed substantial dilatation of the internal jugular vein on the right during straining (Valsalva technique) (Figure 2).

**Figure 3:**
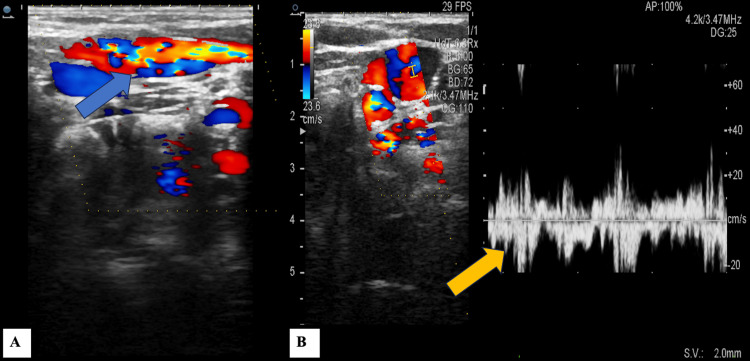
Doppler ultrasound of the neck showing turbulent colour flow in the dilated internal jugular vein (blue arrow) on the right side with no evidence of thrombus formation in (A) transverse view and (B) with spectral waveform (yellow arrow)

**Figure 4:**
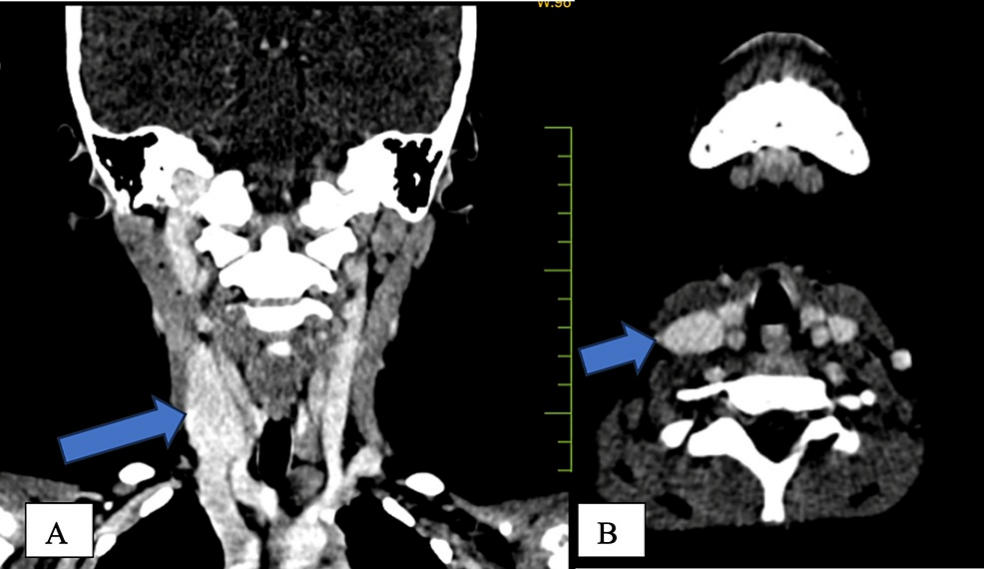
Contrast-enhanced CT scan of the neck in (A) coronal and (B) axial planes which reveals dilatation (blue arrow) of the right internal jugular vein with no evidence of filling defect within the vein

Doppler ultrasound demonstrated colour aliasing, which denotes turbulent flow within the vessel (Figure 3). The patient was also advised to undergo CT (Figure 4), which confirmed the ultrasound findings. Since the swelling was not associated with any complaints, the parents were, therefore, counselled to monitor the patient regularly for any symptoms.

## Discussion

The most likely diagnosis in healthy kids presenting with a soft lump in the neck that only manifests when straining is jugular phlebectasia. Even though there are various other causes for neck masses, only few of them, namely cysts arising from the superior mediastinum, branchial cleft cyst, laryngocele, and jugular phlebectasia, have a tendency to enlarge in size when the patient performs the Valsalva technique [6]. JVP, also known as venous aneurysm or ectasia, refers to a dilatation of the jugular vein. This condition presents as a cystic lesion which can be compressed in the neck and is triggered by the Valsalva manoeuvre [7]. Possible causes of jugular phlebectasia include idiopathic, anatomical, congenital, mechanical, or traumatic origins. JVP is mostly seen in children, occurs more commonly in males than females, and is seen on the right rather than the left side of the neck. The right innominate vein lies in close relation with the apical pleura on the right side, which makes the right jugular vein more prone to dilatation. Consequently, any rise in intrathoracic pressure could be directly transmitted to the right internal jugular vein [8].

JVP is a benign condition and is usually asymptomatic. As a cystic hygroma, cavernous hemangioma, superior mediastinal mass, laryngocele, branchial cyst, and pulmonary apex hypertrophy all present as swelling, which is cystic in nature, JVP should be distinguished from the above-mentioned neck swellings [9]. From chest radiography images, a mediastinal tumour or laryngocele can be ruled out. B-mode ultrasonography and Doppler imaging together are diagnostic and demonstrate colour aliasing, as in our example.

JVP that is asymptomatic must be monitored carefully and does not require any intervention. Our patient is being monitored and is currently symptom-free. Typically, aesthetic considerations or the existence of complications call for surgical treatments. Balik et al. reported a case of jugular phlebectasia with thrombosis and suggested removal of the involved segment surgically without delay in order to prevent further complications [10]. Nevertheless, there is a substantial surgical risk involved with this kind of procedure, which includes injury to adjacent vessels such as the carotid artery, a nerve such as the tenth, eleventh or twelfth cranial nerve or, the phrenic nerve, the subclavian vein or the brachial plexus if the mass extends deeper. Other complications of surgery include an air embolism or venous thrombus formation [11].

## Conclusions

Children presenting with swelling in the neck need to be carefully evaluated. Every time a child exhibits a neck mass that has gotten larger from exercises that raise intrathoracic pressure, such as the Valsalva technique, the diagnosis of JVP should be taken into account. Congenital internal jugular phlebectasia is a rare entity. The diagnosis must be confirmed by imaging. Surgery is only indicated in the presence of complications.
